# EHMTI-0056. Self-medication of headache: identification of subgroups of patients through cluster analysis

**DOI:** 10.1186/1129-2377-15-S1-D46

**Published:** 2014-09-18

**Authors:** E Mehuys, K Paemeleire, G Crombez, T Van Hees, T Christiaens, L Van Bortel, I Van Tongelen, JP Remon, K Boussery

**Affiliations:** 1 Pharmaceutical Care Unit, Ghent University, Ghent, Belgium; 2 Department of Neurology, University Hospital Ghent, Ghent, Belgium; 3 Department of Experimental Clinical and Health Psychology, Ghent University, Ghent, Belgium; 4 Department of Clinical Pharmacy, University of Liège, Liège, Belgium; 5 Department of Family Medicine and Primary Health Care, Ghent University, Ghent, Belgium; 6 Heymans Institute of Pharmacology, Ghent University, Ghent, Belgium

## Introduction

We have previously shown that medication overuse is prevalent among individuals self-medicating regular headache.

## Aims

In this study we evaluated self-medicating headache patients from a broader perspective, exploring the interplay between headache and concomitant pain conditions, pain-related disability and pain medication use. Identification of subgroups of patients could be helpful to tailor intervention strategies.

## Methods

A hierarchical cluster analysis was used to group 1021 self-medicating headache patients according to their (1) sociodemographics, (2) pain characteristics, (3) pain-related disability and (4) pain medication use. Patients were recruited in 202 Belgian community pharmacies and fulfilled the following inclusion criteria: aged ≥18 years, purchasing an over-the-counter systemic analgesic, experiencing pain ≥1 full day/month and suffering from headache.

## Results

Three subgroups were identified. Group 1 comprised patients with low socioeconomic status, low self-rated health, on average four concomitant pain conditions, high pain frequency, high disability, and high rates of medication overuse. Group 2 included older patients with a mean of two other pain syndromes, and low disability but high pain intensity. Group 3 comprised young highly-educated patients diagnosed with migraine, having on average one concomitant pain condition, low pain frequency, low disability but high pain intensity, and low rates of medication overuse.

## Conclusions

We have identified three subgroups in a large sample of individuals self-medicating headache. The marked differences across the three groups stress the importance of a holistic assessment of headache patients and the need for tailored strategies to reduce the risk of medication-overuse headache in primary care.

No conflict of interest.

